# Definitions and Epidemiology of Endodontic Infections

**DOI:** 10.1007/s40496-017-0161-z

**Published:** 2017-11-08

**Authors:** I. F. Persoon, A. R. Özok

**Affiliations:** 1 Department of Preventive Dentistry, Academic Centre for Dentistry Amsterdam (ACTA), University of Amsterdam and Vrije Universiteit Amsterdam, Amsterdam, The Netherlands; 2 Department of Endodontology, Academic Centre for Dentistry Amsterdam (ACTA), University of Amsterdam and Vrije Universiteit Amsterdam, Gustav Mahlerlaan 3004, 1081 LA Amsterdam, The Netherlands

**Keywords:** Endodontic infections, Epidemiology, Primary apical periodontitis, Posttreatment apical periodontitis, Prevalence

## Abstract

**Purpose of Review:**

This review describes the recent findings from epidemiological studies on endodontic infections. The recent literature was screened for studies on the prevalence of primary apical periodontitis and posttreatment apical periodontitis in various populations.

**Recent Findings:**

Twenty-nine articles reporting data on the prevalence of primary or posttreatment apical periodontitis from all over the world were included. The prevalence of apical periodontitis varied between 7 and 86%, while that of posttreatment apical periodontitis varied between 10 and 62%. The quality of the coronal restoration and the root filling appears to be the major predictors of apical periodontitis.

**Summary:**

The recent findings from epidemiological studies on the prevalence of primary and posttreatment apical periodontitis in various populations are not substantially different from those from earlier years. It appears that the health of periapical tissues has not majorly improved in the general population.

## Introduction

### Definition of Endodontic Infections

The root canal system is, in its healthy and intact state, free from infection. Unlike the oral cavity, the root canal system has no commensal microbiota, and any microorganism detected here can be regarded as a potential pathogen. Once microorganisms find their way into the root canal system, the consequences may vary from a simple reversible pulpitis to the necrosis of the pulpal tissue and eventually formation of a periapical lesion: apical periodontitis. Pulpal necrosis on its own, when no microorganisms are involved, does not necessarily lead to apical periodontitis [1]. Nevertheless, a necrotic pulp, following for example a mechanical or thermal trauma, can hardly maintain its ‘sterile’ status due to the lack of blood circulation and defense mechanisms that could eliminate intruding microorganisms. An infected root canal system is a prerequisite for the formation of apical periodontitis [2]. Apical periodontitis is an inflammatory response of the periapical periodontal tissues to the infected root canal system. A bony lesion is formed as calcified periapical tissues erode, caused by the toxic irritation deriving from the infected root canal and the immune response of the host.

The microorganisms identified within endodontic infections are a selection from the commensal microbiota of the oral cavity. The ecological conditions select for a certain microbiota. In a healthy oral ecosystem, microbiota live in homeostasis with the host. When the conditions change, for example by a diet rich in carbohydrates, certain microorganisms can dominate and cause disease. Dental decay may create access to a new niche: the root canal system. Since the ecological conditions are different from the oral cavity, microorganisms will be selected and establish a new microbial community. Although advancing analytical techniques have uncovered an immense diversity and variance of the microbiota within infections, a pattern according to the disease activity can be drawn: symptomatic versus asymptomatic [3]. The microorganisms that contribute the most to endodontic infections are bacteria, both in biomass and diversity [4]. Although *Lactobacillus*, *Actinomyces*, and *Streptococcus* are among the most abundant genera detected in root canals associated with apical periodontitis [5], regarding the aseptic nature of the root canal system in its healthy and intact state, any bacteria present can be regarded as an endodontic pathogen. Still, fungi [4], viruses [6], and archaea [7] can be detected within root canal infections and periapical lesions. Despite their low numbers, they might contribute significantly to the composition and virulence of the overall microbiota and to the host response. Such influence could lead to persistence and symptomology of the apical periodontitis.

### Possible Effect on Epidemiological Studies of the Diagnosis of Apical Periodontitis and the Definition of Its Severity

Symptomatic apical periodontitis is characterized by the inflammation of the periapical tissues, generating clinical symptoms including a painful response to biting or percussion or tenderness with palpation. Depending on the phase of the disease, radiographic changes may or may not be detectable [1]. Asymptomatic apical periodontitis can, however, be solely diagnosed by the presence of radiographic changes, namely a periapical radiolucency created by the bony lesion on the radiograph.

The most commonly used radiographic detection methods for a periapical lesion are conventional or digital periapical radiography, panoramic radiography or, most recently, cone-beam computed tomography (CBCT). Various degrees of diagnostic accuracy for each one of the abovementioned methods have been reported. In the premolar and mandibular molar regions, significantly more periapical lesions can be detected on conventional periapical radiographs than on panoramic radiographs [8].

Due to its three-dimensional nature, the CBCT stands out from the rest [9, 10]. The diagnostic accuracy of CBCT in detecting apical periodontitis is significantly higher compared to digital periapical radiography [10] or panoramic radiography [11]. The possibility of false-negative diagnosis is, therefore, much higher when conventional two-dimensional radiography is used. This may pose a challenge for the diagnosis of the disease, which subsequently may have an impact on the findings of the studies on its prevalence leading to an underestimation. CBCT, on the other hand, has its own limitations with regard to epidemiological studies. Concerning the relatively high effective dose for CBCT and the as low as reasonably achievable (ALARA) principle, when a CBCT scan is made to diagnose apical periodontitis, it is recommended to use limited volume CBCT, in which the field of view (FOV) is limited to a volume smaller than the jaws and suitable for imaging only a few teeth [12]. This may negatively impact studies on the prevalence of apical periodontitis. When a small FOV is used, only a limited part of the dentition is evaluated, and the information about the prevalence of the disease in the rest of the dentition remains censored. From an epidemiological point of view, data collection only on the tooth level, instead of on a subject level, seems less relevant.

Not only the detection of periapical disease is challenging but also the definition of its severity. There seems to be little consensus on the degree of bone destruction at which a bony lesion can be defined as apical periodontitis. Several indexing methods have been introduced for the assessment of the severity of apical periodontitis, each with its limitations. The most commonly used method is the periapical index (PAI) [13], and it is reported to have excellent observer agreement [14]. The PAI scoring system aims to assess the severity of apical periodontitis on dental radiographs with the use of a 5-score scale, where score 1 represents healthy periodontium and scores 2 to 5, respectively, represent periapical disease increasing in severity [13]. This scoring system, however, is not based on direct histopathological examination, but on a simplified modification of the radiographic method of interpretation based on the histopathological examination executed by Brynolf [15]. Since Brynolf used only upper incisors in her study [15], the reliability of the PAI system in premolar or molar regions is unclear.

Another limitation of the PAI system and any other scoring system based on conventional or digital radiographs is related to the two-dimensional nature of these radiographs. While it is, to a certain extent, possible to measure the diameter of the lesion in the mesial-distal direction on a two-dimensional radiograph, its diameter in the buccal-lingual direction remains unmeasurable. The surrounding anatomical structures and the thickness of the bone can further obscure the lesion on the radiograph. This is an even greater problem for multirooted teeth.

There is no clear consensus on the description of apical periodontitis in epidemiological studies using the PAI system screened in this review. While some studies accepted only a PAI score of 1 as healthy apical periodontium [16, 17], many others included a PAI score of 2 in the healthy category as well [18–30, 31•] or even a PAI score of 3 [32].

### The Incidence and Prevalence of Apical Periodontitis

Unfortunately, studies into new diagnoses of endodontic infections per time period, the incidence, are not feasible, since the detection of the onset of the periapical disease is nearly impossible. The time needed for apical periodontitis to become visible on the radiograph is relatively long and depends on several factors. A substantial amount of trabecular bone mineral loss or the erosion of the cortical bone is needed for the lesion to become radiographically visible [33]. Therefore, most studies on the epidemiology of endodontic infections focus on their prevalence, where the proportion of a population with apical periodontitis is estimated based on examination of a sample of the population of interest.

### Recent Findings on the Prevalence of Apical Periodontitis

This review identified relevant studies using a search of the MEDLINE electronic database via PubMed and from the reference list of relevant articles. The search strategy, limited to a period from January 2014 to May 2017, identified the terms ‘apical periodontitis’ and ‘epidemiology’ or ‘prevalence’ and generated 103 hits. Only 51 of these titles were actually related to the search terms. After screening these abstracts, 45 relevant articles were selected for full-text reading. Twenty-nine articles reporting cross-sectional data of epidemiological studies met the inclusion criteria (Table 1). Studies using tooth as the unit of estimation were included only if the data were applicable to subject level or the general population. Outcome studies were excluded, since these only assess the targeted root-filled teeth. Review articles where the abovementioned criteria were not met or if it was not possible to extract this information were excluded as well. The current review defines teeth associated with apical periodontitis and without a root filling as teeth with primary apical periodontitis, and root-filled teeth associated with apical periodontitis as teeth with posttreatment apical periodontitis. Since not every study reported data on the prevalence of primary or posttreatment apical periodontitis separately, in the following part of the text, the term ‘apical periodontitis’ refers to a total of primary and posttreatment apical periodontitis.

**Table 1:** Characteristics and findings of epidemiological studies on apical periodontitis (AP)

											Patient level								Tooth level									
											Root-filled teeth		AP		Primary AP		Post-treatment AP		All teeth	Root-filled teeth		AP		Primary AP		Post-treatment AP		
Publication date	Study date	Country of study	Type of study	Detection method	Index	Systemic condition	Patients (N)	Men (N)	Women (N)	Age range (years)	N	(%)	N	(%)	N	(%)	N	(%)	N	N	(%)	N	(%)	N	(%)	N	(%)	Reference
2014	2007-2008	Sweden	CS	OPT, CE	NR	NR	440	NR	NR	20-89			186	(42)	108	(25)			11876	678	(6)			140	(1)			[34]
2014	2009-2012	UK	CS	CBCT	De Moor [35]	NR	245^a^	128	117	18-86									3595	171	(5)	209	(6)	128	(4)	81	(47)	[36]
2014	2012	UK	CS	OPT	De Moor [35]	NR	136	63	73	16-65+			67	(49)					3396	115	(3)	138	(4)	94	(3)	44	(38)	[37]
2014	NR	Brazil	CS	PR	PAI	Institutionalized elderly	98	41	57	60-94	48	(49)	42	(43)					942	126	(13)	114	(12)	32	(4)	82	(65)	[22]
2014	NR	Serbia	CS	OPT	PAI	NR	153	64	89	18-60	120	(78)							3526	438	(12)					227	(52)	[21]
2014	2008-2009	Denmark	LONG	PR	PAI	NR	327	NR	NR	20-63 at start										350						147	(42)	[24]
2014	2009-2011	Turkey	CS	CBCT		NR	276	142	134	15-72										522						238	(46)	[38]
2014	2009-2010	Brazil	CS	PR	PAI	Coronary artery disease	67	38	29	mean 64			34	(51)														[29]
						Healthy	36	14	22	mean 57			9	(25)														
2014	2004-2012	South Korea	CS	PR	Strindberg [39]	NR	590	265	325	NR										1030						421	(41)	[40]
2015	2013	India	CS	OPT	PAI	NR	1340	NR	NR	18+			865	(65)					30098	1234	(4)	1759	(6)	1297	(4)	462	(37)	[30]
2015	1983-2003	Sweden	RCS	PR, OPT	PAI	NR	788	417	371	20-70										2634						594	(23)	[20]
2015	2012-2013	France	CS	CBCT	CBCTPAI	NR	100^a^	47	53	18-88									2368	431	(18)	204	(9)	28	(1)	176	(41)	[41]
2015	NR	Spain	CS	OPT	Halse and Molven [42]	Post-menopausal osteoporosis	48	none	48	59-68			12	(25)														[43]
						Healthy	27	none	27	59-68			2	(7)														
2015	2009-2011	Nigeria	CS	PR	PAI	NR	285	133	152	18-60			212	(74)					8094	287	(4)	586	(7)	475	(6)	111	(39)	[19]
2015	2009-2011	Nigeria	CS	PR	PAI	NR	756	414	342	20-60+	463	(61)	508	(67)					21468	2625	(12)	3083	(14)	2015	(11)	1068	(41)	[18]
2015	2011-2013	Spain	CS	OPT	PAI	Diabetes mellitus	83	41	42	18+	27	(33)	52	(63)	44	(53)	8	(10)										[23]
2016	2012	Saudi Arabia	CS	OPT	PAI	NR	193	81	112	18-65+										324						190	(59)	[16]
2016	2008-2009	Turkey	CS	CBCT	PAI	NR	748	336	412	15-65										147						93	(63)	[17]
2016	2013-2015	Denmark	CS	OPT	De Moor [35]	Liver cirrhosis	110	71	39	39-82			51	(46)									(4)					[44]
2016	2006-2008	Finland		OPT, CE	PAI	Coronary artery disease	467	254	254	NR	316	(68)	342	(73)					10163	1026	(10)	172	(2)^b^	84	(1)^b^	88	(9)^b^	[32]
2016	2013	Croatia	CS	PR, OPT	PAI	Smoking	108	82	177	mean 40	89	(82)	93	(86)	26	(24)	67	(62)										[25]
						Non-smoking	151				119	(79)	118	(76)	25	(17)	93	(62)										
2017	2010-2012	Saudi Arabia	CS	OPT	Other	Mixed	926	540	386	16-55+									25028	1541	(6)	1559	(6)	942	(4)	617	(40)	[45]
2017	2000-2001	Finland	CS	OPT	Other	NR	5335	47%	53%	30-95	3095	(58)		(27)		(10)		(39)	120635	7986	(7)							[46]
2017	NR	USA	CS	PR, OPT	PAI	Rheumatoid arthritis	131	19	112	22-83	82	(63)	65	(50)					3260	203	(6)	129	(4)	80	(3)	49	(24)	[27]
						Healthy	131	19	112		77	(59)	71	(54)					3395	192	(6)	120	(4)	61	(2)	59	(30)	
2017	2013-2014	Austria	CS	OPT	PAI	NR	1000	430	570	19-91			605	(61)					22586	2504	(11)	1454	(6)	388	(2)	1066	(43)	[28]
2017	2012-2015	Italy	CS	PR, OPT, CE	PAI	Inflammatory bowel diseases	110	49	61	mean 46				(64)														[47]
						Healthy	110	53	57	mean 41				(59)														
2017	2015	Jordan	CS	OPT	PAI	Diabetes mellitus	145	71	74										3111	130	(4)	409	(13)	373	(13)	36	(28)	[26]
						Healthy	146	75	71										3127	57	(2)	372	(12)	361	(12)	11	(20)	
2017	2008	Australia	CS	OPT	PAI	NR	695	292	403	10-88	138	(20)	179	(26)	88	(13)	91	(13)	16936	284	(2)	325	(2)	207	(1)	118	(42)	[31]
2017	2013	Belgium	CS	CBCT	De Moor [35]	NR	631^a^	267	364	mean 45									11117	1357	(12)	656	(6)	212	(2)	444	(33)	[48]

Percentages on patient level were calculated by dividing the number of individuals with one or more AP by the total number of participants. Percentages on tooth level were calculated for root-filled teeth by dividing the number of root-filled teeth by the total number of teeth, for AP by dividing the number of teeth with AP by the total number of teeth, for primary AP by dividing number of teeth with primary AP by the total number of non-root-filled teeth, for post-treatment AP by dividing the number of teeth with post-treatment AP by the total number of root-filled teeth

NR: not reported; CS: cross-sectional; LONG: longitudinal; RCS: repeated cross-sectional; OPT: orthopantomography; CE: clinical examination; CBCT: cone-beam computed tomography; PR: periapical radiography; PAI: periapical index;

^a^scans; ^b^ PAI 1+2+3 =healthy

Based on the included articles, the lowest prevalence of apical periodontitis on a patient level, meaning one or more periapical lesions per patient, was 7% in an otherwise healthy Spanish population [43], and the highest was 86% in a Croatian population of smokers [25] with a median of 52.5%. The corresponding percentages for the prevalence of primary apical periodontitis, respectively, were 10% in a Finnish population [46] and 53% in a Spanish population [23] with a median of 20.5%. The lowest prevalence of root-filled teeth on a patient level was 20% in an Australian population [31•], and the highest was 82% in a Croatian population of smokers [25] with a median of 61%. The lowest prevalence of posttreatment apical periodontitis on a patient level was 10% in a Spanish population [23] and 62% in a Croatian population [25] with a median of 39%.

The prevalence rates on tooth level were lower for each condition. The prevalence of apical periodontitis ranged from 2% in a Finnish population [32] to 14% in a Nigerian population [18]. The median was 6% on a total of 304,721 teeth, which compares well to the 5% found in a previous systematic review and meta-analysis [49••]. This review was on studies from 1987 till 2009 on a total of 300,861 teeth. The prevalence of primary apical periodontitis ranged from 1% in Finnish [32], French [41•], Swedish [34], and Australian [31•] populations to 13% in a Jordanian population of diabetics [26]. The median was 3.5%, which is similar to the 2% from the previously mentioned meta-analysis [49••]. The slightly lower rate might be caused by a more systematic inclusion and also inclusion of studies from mainly well-developed countries. The prevalence of root-filled teeth ranged from 2% in an Australian population [31•] to 18% in a French population [41•]. The median was 6%, which is in the same range as the 10% from the previously mentioned meta-analysis [49••]. The lower prevalence of root-filled teeth in the more recently published studies (published from 2014) might indicate a limited access to oral healthcare, which is likely when studies are done in less-developed countries [30]. The prevalence of posttreatment apical periodontitis ranged from 20% in an otherwise healthy Jordanian population [26] to 65% in a Brazilian population [22]. The median was 41%, which is similar to the 36% found in the previously mentioned meta-analysis [49••]. Overall, the prevalence rates seem similar to rates in not so recent studies (published prior to 2014), even though more recent studies (published from 2014) are done on samples from less-developed countries or individuals with access to newer treatment techniques.

#### Gender

The effect of gender on apical periodontitis remains ambiguous. Studies on Swedish [20], Nigerian [19], Danish [44], or Belgian [48] populations reported no significant difference in the prevalence of apical periodontitis between men and women. However, a significantly higher prevalence of apical periodontitis in men than in women was revealed in Turkish [38], Finnish [46], or Austrian [28] populations, while in Scottish [36], Jordanian [47], or Saudi Arabian [45] populations, the prevalence of apical periodontitis was higher in women than in men. It is quite challenging to explain the difference between the outcome of these studies. Research suggests that women perceive more pain [50] and are more likely to seek treatment for orofacial pain [51], possibly at an earlier, more treatable stage of the disease. Also, cultural characteristics of a population may affect the frequency a man or a woman seeks dental care. Since the results are ambiguous, the effect of gender on apical periodontitis remains unresolved.

#### Quality of the Root Filling or the Coronal Restoration

Both the quality of the coronal restoration and the quality (length or the density) of the root filling are reported to be major predictors of posttreatment apical periodontitis. There is a negative correlation between the quality of the coronal restoration and the prevalence of posttreatment apical periodontitis [16, 17, 20, 28, 30, 40]. Similarly, when the root canal is filled too short of the root apex, the root filling is extruded into the periapex, or the root filling contains voids, the prevalence of posttreatment apical periodontitis increases [16, 17, 20–22, 24, 28, 30, 37, 40, 48].

#### Systemic Conditions

An increasing number of studies are conducted to reveal a possible bidirectional association between the apical periodontitis and the systemic health. This means that a systemic condition can be aggravated by apical periodontitis and, vice versa, that systemically affected individuals are more prone to apical periodontitis. Thus far, limited studies are available on the effect of systemic disease on endodontic outcome [52••, 53–55]. Besides, the evidence is of limited quality and indefinite, which demands that the found associations are to be interpreted with caution [52••]. In order to detect indisputable bidirectional associations between oral disease and the systemic conditions, there is an urgent need for the application of stringent criteria on research reporting [56].

Individuals with uncontrolled diabetes mellitus are reported to have significantly more periapical lesions in comparison to controls with well-controlled diabetes mellitus (23 vs. 49% [23], 9 vs. 18% [26]). Also, individuals with diabetes mellitus are 1.4 times more likely to have root-filled teeth with apical periodontitis than healthy controls [53]. Apical periodontitis might decrease glycemic control, aggravating diabetes. In turn, diabetes might limit the host’s inflammatory response to combat the endodontic infection. In women suffering from postmenopausal osteoporosis, low bone density is reported to be associated with a higher prevalence of apical periodontitis [43]. An association between the prevalence of apical periodontitis and coronary artery disease is reported, where the inflammatory burden of the endodontic infection might be contributing to the systemic condition [29, 32]. Individuals with liver cirrhosis and periapical lesions also had higher C-reactive protein levels and a higher prevalence of cirrhosis-related complications such as ascites, hepatic encephalopathy, and variceal bleeding [44]. Women suffering from inflammatory bowel disease are reported to have more teeth with apical periodontitis and with more severe bone destruction. The prevalence of apical periodontitis was even higher when these women were treated with immunomodulators [47]. Regarding the prevalence of apical periodontitis, there was no significant difference between individuals with rheumatoid arthritis [27] and their healthy counterparts.

#### Smoking

Smoking is reported to be one of the strongest predictors of apical periodontitis in a Danish [44] and in a Nigerian population [19]. A Croatian population of smokers had two times more apical periodontitis than the never-smokers did [25]. Smoking might negatively affect the periodontal bone and is associated with dental caries, one of the main causes of apical periodontitis.

#### Age

Several studies described age as a predictor for apical periodontitis [19, 44]. The findings of a systematic review and meta-analysis of cross-sectional studies on the prevalence of apical periodontitis and root-filled teeth in persons 65 years of age or older conducted in 2016 do not deviate substantially from the findings of the studies included in this review [57••]. That review confirmed that the prevalence of both apical periodontitis and root-filled teeth increases with age. With aging, not only the comorbidity increases but also the immune system deteriorates. Additionally, oral disease experience in the elderly is higher than younger adults [58]. This might be an important confounder when considering age as a predictor for apical periodontitis.

## Conclusions

Although there seems to be an increase in recent years in studies from less-developed countries and newer detection techniques for periapical disease, such as CBCT, are introduced, the findings from epidemiological studies on the prevalence of primary and posttreatment apical periodontitis in various populations are not substantially different from those from earlier years. It appears that the health of periapical tissues has not majorly improved in the general population, despite technological progress of endodontic treatment and increasing access to healthcare worldwide.

Of all the studied possible predictors, the quality of the coronal restoration and the root filling are the most consistently reported risk factors for apical periodontitis. To identify all possible risk factors efficiently, the design and reporting of epidemiological studies on apical periodontitis should be standardized and improved.
